# The Effectiveness of Triple Dose Albendazole in Treating Soil Transmitted Helminths Infection

**DOI:** 10.1155/2019/6438497

**Published:** 2019-02-04

**Authors:** Saleha Sungkar, Kartika Q. Putri, Muhammad I. S. Taufik, Meutia N. Gozali, Pratiwi Sudarmono

**Affiliations:** ^1^Department of Parasitology, Faculty of Medicine, Universitas Indonesia, Jakarta, Indonesia; ^2^Department of Microbiology, Faculty of Medicine, Universitas Indonesia, Jakarta, Indonesia

## Abstract

Soil transmitted helminths (STH) infection is a major health problem in tropical countries such as Indonesia. Albendazole is an effective and widely used anthelmintic agent to treat STH; however, it is not effective towards* T. trichiura *and its effectiveness varies between populations. Hence, we conducted a study to determine the effectiveness of triple dose albendazole in children of Perobatang village, Southwest Sumba, Indonesia. A pre-post study was carried out in Perobatang village on July 2016. Children aged 1-15 years old were enrolled in the study and asked to collect stool samples which were then examined using Kato-Katz method. The children infected with STH were given albendazole 400 mg for three consecutive days. From 246 subjects examined, 192 (78%) were positive for any STH consisting of* T. trichiura* (64%),* A. lumbricoides* (60%), and hookworms (10%). After treatment, the prevalence of STH decreased significantly (McNemar test, p<0.001) to 27%,* T. trichiura* 25%,* A. lumbricoides* 2%, and hookworm 0%. Cure rate for* T. trichiura, A. lumbricoides, *and hookworms was 61%, 97%, and 100%, respectively. Significant decrease of eggs per gram of feces was found in all STH (Wilcoxon test, p value <0.001 for* A. lumbricoides* and* T. trichiura*, p value = 0.027 for hookworms); egg reduction rate for* T. trichiura *was 91%*, A. lumbricoides *was 100%, and hookworms was 100%. In conclusion, triple dose albendazole is effective in controlling STH in children of Perobatang village, Southwest Sumba, Indonesia.

## 1. Introduction

Soil transmitted helminths (STH) infection is a public health problem especially among rural areas in tropical countries. The most common STH are* Ascaris lumbricoides, Trichuris trichiura, *and hookworms [1, 2]. The highest prevalence of STH occurs in areas where sanitation is inadequate and clean water is scarce such as Southwest Sumba, East Nusa Tenggara, Indonesia. Sungkar et al. [3] reported that in Kalena Rongo village, Southwest Sumba, the prevalence of intestinal parasitic infection was 95.5% [*A. lumbricoides *(65.8%),* T. trichiura *(60.4%), hookworms (53.5%)].

The burden of STH is mainly due to its chronic and insidious impact on the health and quality of life rather than mortality. STH infections adversely affect child growth and development, nutritional status, and cognitive capacity [4].

The best method to interrupt transmission of STH is by providing an effective and efficient sanitation. However, in endemic countries the resources required to sustain infrastructure are rarely available, thus eliminating morbidity is more feasible. WHO [4] recommends periodic administration of single dose albendazole 400 mg or mebendazole 500 mg to control STH in populations at risk including pre-school and school-age children. However, treatment of STH using those regimen of anthelmintic possesses low cure rate for* T. trichiura* (34%) [5]. The effectiveness of albendazole can be increased by giving a dose of 400 mg for three consecutive days (triple dose), yet the effectiveness of albendazole varies between populations. Therefore, a study is required to investigate the effectiveness of triple dose 400 mg albendazole in treating STH.

## 2. Method

### 2.1. Study Area

Southwest Sumba was ranked as the second poorest district in East Nusa Tenggara. Poor hygiene is mainly due to the difficulty in accessing clean water which can only be obtained from artesian well, located far from residential areas. Hence, clean water is only used sparingly for drinking and cooking, rarely for bathing and hand hygiene. In addition, people are used to free defecation as their traditional houses are ill-equipped with latrines and sanitation.

Perobatang village is a small rural community located 70 km from the capital of Southwest Sumba district (Tambolaka). In 2015, it has an area of 4360 hectares with 315 families and a total population of 1459 (498 children, 231 early-adults, and 630 adults aged >40 years). The predominant occupations are fishermen and farmers. There is no primary health centre (PHC) facility in the village. The nearest PHC is located almost 3 km away in Bondokodi subdistrict. The village was chosen due to high risk factors of STH infection (free defecation, poor hand washing habit, inaccessible clean water sources, low socioeconomic status), and the lack of mass drug administration (MDA) program with albendazole conducted in the village.

### 2.2. Study Design

This study used pre- and post-study design conducted in Perobatang village, Southwest Sumba, Indonesia, in July 2016.

### 2.3. Study Subjects

The subjects were all children in the village, aged 1-15 years, who got parental permissions; children with fever were excluded.

### 2.4. Data Collection

The village chief's residence was converted into a makeshift clinic. Baseline data was collected on the first week of July 2016 prior to treatment and followed up two weeks after treatment (to study the effectiveness of anthelmintic, the best interval between treatment and resampling should be 10-14 days).

On the first day, the subjects and their parents were given informed consent. Demographic characteristics were obtained from the parents or the subjects. They were also given explanation on procedure of collecting the fecal samples.

To collect the fecal samples, thumb-sized feces were put into 10 cc pot. Stool pots were labeled with the identity of the subjects: name, code data, gender, and age. The next day, the subjects were asked to return the samples to the researchers. Kato-Katz thick smears method was prepared from each sample [6] and the samples were examined under a light microscope (40X magnification). Number of eggs per gram (EPG) of the fecal sample was recorded for each species of worms. If the result was negative in the first smear, the examination was repeated for three times by the same examiner.

### 2.5. Study Treatment

The study used WHO guideline for treatment using albendazole [4]. Subjects older than two years old infected with STH were given 400 mg albendazole while those between one and two years old were given 200 mg albendazole. Albendazole was given for three consecutive days (triple dose) and subjects were asked to swallow the tablet in front of the researchers. To assess safety and side effects, patients and/or the parents were questioned for any clinical complaints after taking the drugs.

### 2.6. Outcome Assessment

To determine the effectiveness of albendazole triple dose, we compared the cure rate (CR) and eggs reduction rate (ERR) calculated using the formula below.

(1) CR=number  of  subjects  infected  with  STH  who  were  curednumber  of  infected  subjects  who  were  treated×100%ERR=mean  EPG  before  treatment−mean  EPG  after  treatmentmean  EPG  before  treatment×100%

We calculated the total CR and ERR along with CR and ERR for each species of worms found in the feces samples.

### 2.7. Data Analysis

The treatment was considered effective if CR was >90% and ERR was >90%. To obtain the prevalence of STH before and after treatment, data was analyzed using McNemar test in SPSS version 20 and the difference of EPG before and after treatment was analyzed using Wilcoxon test. For all tests, a p-value of 0.05 was considered the limit of statistical significance and 95% confidence intervals (CIs) were calculated as appropriate.

### 2.8. Ethical Clearance

This study has obtained ethical approval from Ethical Committee of Faculty of Medicine, Universitas Indonesia (Ref. No. 771/UN2.F1/ethic/2015).

## 3. Results

### 3.1. Subjects Characteristics

All children (498 children) in the village were recruited. Among those children, 246 returned the pot with the samples and were all examined for STH infection. A total of 192 children were positive for STH and treated at the start of the study.  Table 1 shows that most subjects were between 5 and 15 years old (72%) and the prevalence of STH was 78% consisting of* T. trichiura *(64%),* A. lumbricoides* (60%), and hookworm (10%).

### 3.2. Cure Rate and Egg Reduction Rate

Table 2 shows that two weeks after the treatment, the prevalence of* A. lumbricoides, T. trichiura*, and hookworms decreased significantly (McNemar test, p<0.001). The CR of hookworm and* A. lumbricoides* is more than 90% while that of* T. trichiura *is less than 90%. After treatment, the EPG for the three types of worms decreased significantly (Wilcoxon test, p<0.001 for* A. lumbricoides *and* T. trichiura*, p=0.027 for hookworms, Table 3).

## 4. Discussion

The strategy for STH control in endemic areas focuses on morbidity control through large scale administration of single dose anthelminthics to at-risk populations, especially school-age children [7, 8].

In this study, we show the effectiveness of triple dose albendazole in reducing the prevalence of STH. Prior to the intervention we found that the prevalence of the three types of worms was very high, especially among school-aged children as they spend more time outdoors compared to the younger children. Smaller children tend to spend more time carried by mothers, hence limiting their contact with soil. Prevalence of each species of worm is more than 50%, but not in hookworms (10%) because the prevalence of hookworm infection is higher in adults compared to children. Adults usually work in the farm where the soil is loose while the children play in the yard of the house that is clay. The clay soil is a suitable factor for the development of life cycles of STH, particularly* A. lumbricoides *and* T. trichiura*, while hookworms prefer sandy soil for the aeration larval development. The warm climate in this area is a supporting factor in the development of STH, especially for embryonation of STH eggs.

Our finding is similar to that of Steinman et al. [5] that reported efficacy of triple dose of albendazole 400 mg compared to single dose albendazole and single dose or triple dose of mebendazole. The efficacy of albendazole is higher compared to mebendazole, except for* T. trichiura*. Triple dose albendazole gave a high CR for* A. lumbricoides *(96.8%) and hookworm (92%); meanwhile for* T. trichiura*, the CR of triple dose albendazole was 56.2% and that of triple dose mebendazole was 70.7% [9].

Randomized controlled trial conducted by Adegnika et al. [10] compared the albendazole single dose and repeated doses in the treatment of* A. lumbricoides, T. trichiura,* and hookworm. Adequate cure rates and egg reduction rates above 85% were found with a single dose of albendazole for* A. lumbricoides* infection, 85% and 93.8%, respectively, while two doses were necessary for hookworm infestation (92% and 92%, respectively). However, while a 3-day regimen was not sufficient to cure* T. trichiura* (CR 83%), this regimen reduced the number of eggs up to 90.6%. Treatment led to a reduction of multiple infections and double or triple infections were rare after a treatment with one or two doses

The reduction and the maintenance of low worm burden have an important impact on the health of the community. The first sign of improvement is parasitological with a reduction of heavy infection, then nutritional, with an increase in iron stores, followed by an increase in hemoglobin level and finally by an increase in growth. At least two years of intervention are normally required before an increase in hemoglobin becomes evident and even longer period is required to exhibit improvement in growth. Improved indicators of school effects (school attendance, reenrollment, retention, and achievement) have been observed: iron load and increasing hemoglobin level, improving physical growth and cognitive capacity, educational achievement, and reduced school absence [11].

Without significant environmental and health behavioral improvement, reinfection will occur and may reach the same prevalence and intensity prior to the treatment. Jia et al. [12] found that reinfection will occur in 6-12 months after the treatment, while Appleton et al. [13] reported that within five months there had been a 75% reinfection of* A. lumbricoides*, 71%* T. trichiura* reinfection, and 28% reinfection of hookworm. Total reinfection will return within 12 months if no retreatment is applied.

Since it is difficult to change environmental and health behavioral patterns in Perobatang village, mass drug treatment is required every six months for at least five consecutive years to control STH. Tun et al. [14] conducted a study in Myanmar and found that 400 mg of albendazole every 6 months for seven years succeeded in controlling STH even without changing the subjects' environmental and behavioral practices.

Albendazole is effective in treating* A. lumbricoides *and hookworm but not effective for* T. trichiura.* However, a high ERR on albendazole can reduce environmental contamination with* T. trichiura* eggs, and if mass treatment is carried out for more than 5 years, STH will be eliminated including* T. trichiura*. In areas with high trichuriasis, albendazole should be given in three consecutive days to enhance the elimination of* T. trichiura*.

## 5. Conclusion

Triple dose albendazole is effective in reducing prevalence of* A. lumbricoides, T. trichiura, *and hookworms in tropical remote area with limited clean water access, poverty, and poor education.

## Figures and Tables

**Table 1:** The characteristics of subjects in Perobatang village, 2016.

**Characteristics**	**Any STH**	***T. trichiura***	***A. lumbricoides***	**Hookworm**
**Gender**				
Male, n=114	82 (33%)	67 (27%)	67 (27%)	14 (6%)
Female, n=132	110 (45%)	90 (37%)	81 (33%)	10 (4%)
**Age**				
1 – 4 yo, n=69	51 (21%)	35 (14%)	40 (16%)	3 (1%)
5 – 15 yo, n=177	141 (57%)	122 (50%)	108 (44%)	21 (9%)
**Total (n=246)**	**192 (78**%**)**	**157 (64**%**)**	**148 (60**%**)**	**24 (10**%**)**

**Table 2:** The prevalence of STH and cure rate after intervention.

**Species of Worm**	**Prevalence **		** CR (95**%** CI)**
	**Before**	**After**	
*A. lumbricoides*	148 (60%)	5 (2%)	97% (90–100)
*T. trichiura*	157 (64%)	62 (25%)	61% (53–68)
Hookworms	24 (10%)	0 (0%)	100% (96–100)

**Table 3:** The comparison of EPG before and after intervention.

**Species of Worm**	**Mean EPG** **∗**		**ERR (95**%** CI)**
	**Before**	**After**	
*A. lumbricoides*	8.592	24	100% (99–100)
*T. trichiura*	1.344	120	91% (88–93)
Hookworms	24	0	100% (99–100)

*∗*Average number of eggs per gram of feces.

## Data Availability

The data used to support the findings of this study are included with this article.
